# Anti-Müllerian hormone in relation to adiposity and metabolic indices in polyendocrine metabolic ovarian syndrome

**DOI:** 10.3389/fendo.2026.1975667

**Published:** 2026-09-24

**Authors:** Karolina Kowalczyk, Dominika Gałczyńska, Julia Herman, Marta Czyż, Dariusz Kowalczyk, Agnieszka Drosdzol-Cop, Grzegorz Franik

**Affiliations:** 1 Chair and Department of Gynecology, Obstetrics and Oncological Gynecology, Medical University of Silesia, Katowice, Poland; 2 Department of Endocrinological Gynecology, Faculty of Medical Sciences, Medical University of Silesia, Katowice, Poland; 3 Department of Anatomy, School of Medicine in Opole, University of Opole, Opole, Poland

**Keywords:** AMH, anti-Müllerian hormone, metabolic dysfunction, PCOS, PMOS, polycystic ovary syndrome, polyendocrine metabolic ovarian syndrome, visceral obesity

## Abstract

**Introduction:**

Anti-Müllerian hormone (AMH) is widely used as a marker of ovarian reserve and is also incorporated into the diagnostic assessment of polyendocrine metabolic ovarian syndrome (PMOS). Although PMOS is strongly associated with metabolic dysfunction and abnormal adipose tissue distribution, the relationship between AMH concentrations and adiposity, and especially visceral adiposity remains unclear. This study aimed to evaluate the association between serum AMH levels and selected adiposity and metabolic markers in women with PMOS.

**Methods:**

This cross-sectional study included 1147 women with newly diagnosed PMOS. Serum AMH, sex hormone-binding globulin (SHBG), glucose, insulin, lipid profile, and other hormonal parameters were assessed. Anthropometric measurements included body mass index (BMI), waist and hip circumference, and waist-to-hip ratio (WHR). Percent body fat (PBF) and visceral fat area (VFA) were assessed using bioelectrical impedance analysis. The lipid accumulation product (LAP), cardiometabolic index (CMI), and homeostatic model assessment of insulin resistance (HOMA-IR) were calculated.

**Results:**

AMH concentrations showed very weak inverse correlations with adiposity markers, WHR (*r* = -.05, *p* <.05), VFA (*r* = -.1, *p* <.05), CMI (*r* = -.06, *p* <.05), and LAP (*r* = -.1, *p* <.05), while a positive correlation was observed between AMH and SHBG levels (*r* = .07, *p* <.05). In contrast, LAP was moderately or strongly positively correlated with fasting insulin (*r* = .67, *p* <.05), HOMA-IR (*r* = .67, *p* <.05), PBF (*r* = .76, *p* <.05), and anthropometric measures, and negatively correlated with HDL cholesterol (*r* = -.64, *p* <.05) and SHBG (*r* = -.62, *p* <.05). Principal component analysis (PCA) identified two major metabolic dimensions, predominantly reflecting triglyceride and cholesterol-related variation, which together explained 68.8% of the total variance. AMH showed no correlation with any of them.

**Conclusions:**

In women with PMOS, serum AMH concentrations are negligibly associated with adiposity markers despite strong relationships between these markers and metabolic dysfunction. Therefore, AMH levels should be interpreted primarily in the context of age and reproductive characteristics rather than as a direct marker of visceral adiposity or metabolic risk.

## 1 Introduction

Polyendocrine metabolic ovarian syndrome (PMOS), previously named polycystic ovary syndrome (PCOS), affects one in eight women (1). It is the most common cause of hyperandrogenism, as well as a main cause for ovulatory dysfunction in women treated for infertility. It presents itself clinically in various ways, ranging from endocrine and reproductive symptoms such as irregular menstrual cycles, acne, to hirsutism and excess weight (2). Patients with PMOS are also more at risk of suffering from additional metabolic consequences, including glucose intolerance and diabetes mellitus, metabolic-associated fatty liver disease or dyslipidemia (3).

To be diagnosed with PMOS, it is required to have at least two of the following three criteria: ovulatory dysfunction (rare or lack of ovulation); hyperandrogenism (clinical or/and biochemical); polycystic ovarian morphology or elevated anti-Müllerian hormone (AMH) levels. These criteria apply to adult women. To diagnose adolescents, ovarian morphology or AMH levels are not required (4).

AMH is a polypeptide of the transforming growth factor beta family, produced by the granulosa cells in the ovaries. It is a marker used in the diagnostics of PMOS, as it correlates with the size of the primordial follicle pool. It shows a significant increase in the number of patients with this syndrome. The serum concentration of AMH is also linked to the severity of the PMOS symptoms and might be correlated to metabolic syndrome and its symptoms (5, 6). Serum AMH was first incorporated into the diagnostic framework for PMOS in the 2023 International Evidence-Based Guidelines, where it was introduced as an alternative marker for defining polycystic ovarian morphology in adults (4).

The correlation between serum AMH concentration and metabolic risk has been examined in other studies, with inconclusive results. However, the results clearly show that AMH levels are significantly lower in obese patients with PMOS than in patients with PMOS but with a normal body weight (7).

It has been stated that AMH levels are significantly higher in patients with PMOS, especially when insulin resistance is also present (8). In contrast, other reviews have found no correlation between indicators of insulin resistance and AMH levels (9). The relationship between AMH concentration and body mass index (BMI) has also been examined in several studies, with some reporting a negative correlation (9–11). The correlation between the lipid profile and AMH concentrations in patients with PMOS has also been discussed, with the results being inconclusive - some showing a possible correlation and some stating no significant relationship between them (12–14). Most reviews state that more research should be done to establish the relationship between AMH and metabolic risks in women with PMOS.

Lately, other metabolic indicators, such as PBF (percent body fat), VFA (visceral fat area), LAP (lipid accumulation product), and CMI (cardiometabolic index), have played an important role in assessing metabolic risk.

PBF allows for more precise measurement of adiposity than lean tissue, while BMI is used mainly for population-level monitoring due to its simplicity (15). Furthermore, higher body fat percentage is associated with a greater risk of metabolic syndrome, cardiovascular dysfunction, and mortality among individuals with normal body mass index (16). Visceral Fat Area (VFA) is another very important indicator of metabolic risk, with studies also showing a strong correlation with metabolic syndrome across all BMI categories (17). VFA is measured in square centimeters, with its ideal value less than 100 cm^2^ (18). Both factors can be obtained through bioelectrical impedance analysis (BIA). It is a safe, non-invasive method to measure body composition based on the electrical properties of biological tissues (19). BIA parameters can be used to estimate body composition properties such as fat, fat-free mass, total-body water and its compartments (20). There have been multiple studies evaluating the differences in body composition between women with PMOS and women without this syndrome (21–23). However, most of them did not show significant differences between the two groups (21–23) and did not specifically correlate their findings with AMH serum levels.

While BIA is a valuable tool in both clinical practice and research for assessing body composition, it is not the only available method. Dual-energy X-ray absorptiometry (DXA) enables rapid, non-invasive measurement of fat mass, fat-free mass, and bone mineral density and is considered the reference standard in clinical research. However, due to its high cost and the need for specialized radiological equipment, DXA is not routinely used in clinical settings and was therefore not used in this examination (24).

LAP is an index calculated from waist circumference (WC) and triglyceride (TG) levels and is mainly used to evaluate Metabolic Dysfunction-Associated Fatty Liver Disease (MAFLD) (25). It has also been shown to perform better than BMI in identifying cardiovascular risk (26). CMI defines adiposity based on the triglycerides to high-density lipoprotein cholesterol (HDL) ratio and waist-to-height ratio (WHtR) and has been also used to detect cardiovascular risk factors (27).

Although the topic and relationship between AMH concentration and metabolic dysfunctions have been well discussed, there doesn’t seem to be a clear answer to whether a correlation exists.

This study aimed to evaluate the relationship between the visceral adiposity markers, such as PBF, VFA, LAP, CMI and AMH in PMOS patients.

## 2 Materials and methods

### 2.1 Patients

This cross-sectional single-center study included a cohort of 1147 women aged 18–35 years newly diagnosed with PMOS (previously PCOS) during hospitalization in the Endocrinological Gynecology Department of Medical University of Silesia in Katowice between December 2021 and December 2025. The exclusion criterion for patients in the study was the occurrence of diseases that could cause similar symptoms, such as hyperthyroidism, hypothyroidism, congenital adrenal hyperplasia, androgen-secreting tumors, Cushing’s syndrome, hypogonadotropic hypogonadism, premature ovarian failure, or hyperprolactinemia. Hormonal contraceptives, steroids, antiandrogens, ovulation induction agents and hypoglycemic drugs were withdrawn 3 months before the admission.

Formal approval to use of retrospective data from the patients’ files was obtained from the Bioethics Committee of the Medical University of Silesia, approval number KNW/NWN/0052/KB/268/24. Informed consent was waived because of the retrospective nature of the study. The study was conducted in accordance with the ethical standards of the institutional and/or national research committee and with the 1964 Declaration of Helsinki and its later amendments or comparable ethical standards.

### 2.2 Methods

Due to retrospective character of the research, the diagnosis of PMOS (called PCOS at the time of diagnosis) was established on the presence of at least two out of three Rotterdam Criteria: oligo- or anovulation (irregular cycles < 21 or >35 days), hyperandrogenism (clinical or biochemical: above the normal range for total testosterone, free testosterone, dehydrepiandrosterone sulfate, or androstenedione), and ultrasound assessment of polycystic ovarian morphology (PCOM). Clinical hyperandrogenism included hirsutism, acne, or alopecia. Hirsutism was assessed using the modified Ferriman-Gallwey score. It is a semi-quantitative assessment and involves nine androgen-sensitive body areas (upper lip, chin, chest, upper back, lower back, upper abdomen, lower abdomen, upper arms, thighs), each scored from 1 (minimal terminal hair growth) to 4 (pronounced virilization). The scores across all areas were summed, yielding a total score of 8 or higher, indicating hirsutism (29). Biochemical hyperandrogenism was defined as serum total testosterone >0.481 ng/mL, free testosterone > 4.1 pg/mL, and/or free androgen index >5% (30). Transvaginal ultrasound was performed using Voluson E8 Expert, GE Healthcare, by the same examiner. PCOM was diagnosed when more than 12 antral follicles in at least one ovary were noted or ovarian volume was over 10 ml (28). Patients were classified into different phenotypes. The Rotterdam criteria allow to distinguish four distinct phenotypes based on the presence of oligo- or anovulation, hyperandrogenism, and polycystic ovaries. Phenotype A (classic) oligo- or anovulation, hyperandrogenism, and polycystic ovaries; phenotype B (non-PCOM) - oligo- or anovulation and hyperandrogenism, but normal ovarian morphology; phenotype C (ovulatory) - hyperandrogenism and polycystic ovaries, but regular ovulation; phenotype D (mild) - oligo- or anovulation and polycystic ovaries, but normal androgen levels. AMH was measured in every patient, however, it was not incorporated in the diagnosis.

### 2.3 Measurements

Height, weight, waist and hips circumference were measured in each participant. Waist-hip ratio (WHR) was calculated waist circumference (WC) divided by hip circumference, whereas BMI was calculated as weight (kg) divided by height squared (m^2^). Blood samples were collected between the second and sixth day of the menstrual cycle, to assess hormonal parameters including androgens, sex hormone-binding globulin (SHBG) and AMH levels. Successively, after an overnight fast, glucose, insulin, total cholesterol, HDL cholesterol, LDL cholesterol, and triglycerides. Afterward, oral glucose tolerance test (OGTT) was performed - glucose was measured at 0′ and 120′, and HOMA-IR was calculated by the formula (glucose [mmol/L] × insulin [µU/L])/22.5.

In addition, all examined women underwent routine hormonal testing for the differential diagnosis of PMOS, including measurements of gonadotropins, prolactin, thyroid function, 17-hydroxyprogesterone, androstenedione, and dehydroepiandrosterone.

To assess general and visceral adiposity, several indices were included. PBF and VFA were determined using BIA - the InBody170 body composition analyzer (InBody, Poland). Normal PBF ranged from 18-28%. VFA was reflected as visceral fat rating and its cut-off below 10 corresponded to 100 cm^2^. CMI was calculated using the formula: (Triglycerides [mmol/L] ÷ HDL-C [mmol/L]) × (WC [cm] ÷ height [cm]). Values over 1.14 are interpreted as indicative for elevated cardiometabolic risk (31). LAP was calculated from the following formula (WC [cm] - 58) × Triglycerides [mmol/L]. Its cut-off values vary depending on sex, age, and the specific metabolic or cardiovascular risk being evaluated, but general clinical thresholds typically range from 20–55 cm·mmol/L (32).

### 2.4 Biochemical analyses

Serum glucose, total cholesterol, HDL and LDL cholesterol, and triglycerides were determined by colorimetry (AU 640 analyzer) using Beckman Coulter reagents (Brea, California, USA). Insulin concentrations were determined using the chemiluminescence method (CMIA) using Abbott reagents (Alinity instrument; Chicago, Illinois, USA). Serum concentrations of SHBG and AMH were determined by electrochemiluminescence “ECLIA” using Roche reagents on a Cobas 601 analyzer (Roche Diagnostics GmbH, Mannheim, Germany).

### 2.5 Statistical analysis

The statistical analysis was carried out in the RStudio programme using the R programming language. The descriptive analysis was presented in table form. The normality of the distribution was assessed using histograms and QQ plots generated in RStudio. The correlation analysis was carried out using Spearman’s correlation coefficient. Relationships between variables were assessed using analysis of variance for normally distributed quantitative variables and the Kruskal-Wallis test for non-normally distributed quantitative variables. Benjamini-Hochberg correction was waived, because of very low p-values ​​(i.e. high significance), while the application of the correction has the greatest impact on the results close to the threshold of significance. To determine whether AMH was associated with adiposity markers independently of age, BMI and PMOS phenotype, linear regression models were constructed. Regression results were presented as β coefficients with 95% confidence intervals. A p value <0.05 was considered statistically significant. Principal component analysis (PCA) was then performed, and two extracted factors were included in subsequent analyses.

## 3 Results

1147 women with PMOS participated in the study. The average age was 25.22 ± 4.87 years old. Most patients (n=724) presented with phenotype A, followed by 117 with phenotype B, 177 with phenotype C, and 129 with phenotype D. Anthropometric and metabolic characteristics of participants are presented in the table below (**Table 1**). The average AMH serum concentration in this group was 6.48 ± 4.48 ng/mL, while the means of visceral adiposity indices, such as CMI and LAP, were 0.99 ± 0.67 and 31.24 ± 33.14 cm*mmol/L, respectively. The average percent body fat value was 34.14 ± 9.59%, while the average visceral fat area was 11.73 ± 6.76. These variables were used to evaluate the correlations between AMH serum concentration and adiposity markers in women with PMOS.

**Table 1:** Anthropometric and metabolic characteristics in the study group.

Variable	N	Min	Max	Median	Q1	Q3	Mean	SD
Age (years)	1147	18	35	25	22	28	25.219	4.866
Weight (kg)	1147	39	150	67	59	82	72.422	18.869
Height (m)	1147	144	185	166	162	170	166.044	6.015
BMI (kg/m^2^)	1147	15.23	53.15	24.342	21.349	29.74	26.24	6.532
Waist (cm)	1147	57	145	80	71	92	83.173	15.579
Hip (cm)	1147	76	166	101	95	110	103.629	12.578
WHR	1147	0.55	1.14	0.79	0.74	0.85	0.803	0.084
Triglycerides (mmol/L)	1147	0.2	4.54	0.92	0.69	1.28	1.062	0.532
Cholesterol (mmol/L)	1147	2.16	7.63	4.34	3.83	4.89	4.395	0.793
LDL (mmol/L)	1147	0	4.95	2.35	1.94	2.87	2.429	0.706
HDL (mmol/L)	1147	0.55	15.15	1.44	1.21	1.7	1.491	0.552
SHBG (nmol/L)	1147	5.7	198	45.1	30.5	65.55	51.093	28.631
AMH (ng/ml)	1147	0.2	61.4	5.54	3.52	8.32	6.475	4.483
Insulin 0’ (µU/mL)	1147	0.9	83	7.62	5.335	11.5	9.676	7.362
Glucose 0’ (mmol/L)	1147	3.72	6.71	4.71	4.53	4.93	4.749	0.353
Glucose 120’ (mmol/L)	1147	2.29	14.98	5.99	5.04	7.1	6.237	1.769
PBF [%]	1147	8.4	55.3	34.3	26.65	41.1	34.142	9.593
VFA	1147	1	31	10	6	16	11.728	6.763
Skeletal muscle mass (kg)	1147	15.8	42.3	25.1	22.7	27.8	25.586	4.038
LAP (cm*mmol/L)	1147	-0.704	281.392	18.905	9.642	40.86	31.239	33.135
HOMA-IR	1147	0.149	20.905	1.596	1.107	2.496	2.077	1.671
CMI	1147	0.071	5.311	0.787	0.539	1.258	0.997	0.674

SD, standard deviation; BMI, body mass index; WHR, waist–hip ratio; LDL, low-density lipoprotein; HDL, high-density lipoprotein; SHBG, sex-hormone binding globulin; AMH, anti-Müllerian, PBF–percent body fat body fat, VFA–visceral fat area, LAP–lipid accumulation product, HOMA-IR, Homeostatic Model Assessment of Insulin Resistance, CMI–cardiometabolic index.

### 3.1 AMH in relation to adiposity and metabolic indices

**Figure 1** presents the correlation matrix of analyzed variables (only statistically significant correlations with *p* <.05 are shown). Overall, the correlations between AMH and adiposity markers were negligible, with LAP (*r* = -.1), VFA (*r* = -.1) and WHR (*r* = -.05) showing negative statistically significant correlations with the hormone level. Additionally, AMH showed negative correlation with fasting glucose (*r* = -.09), BMI (*r* = -.13) and skeletal muscle mass (*r* = -.12), and positive association with SHBG levels (*r* = .07). The correlations among the metabolic parameters themselves were much stronger, reflecting their shared physiological components.

**Figure 1:**
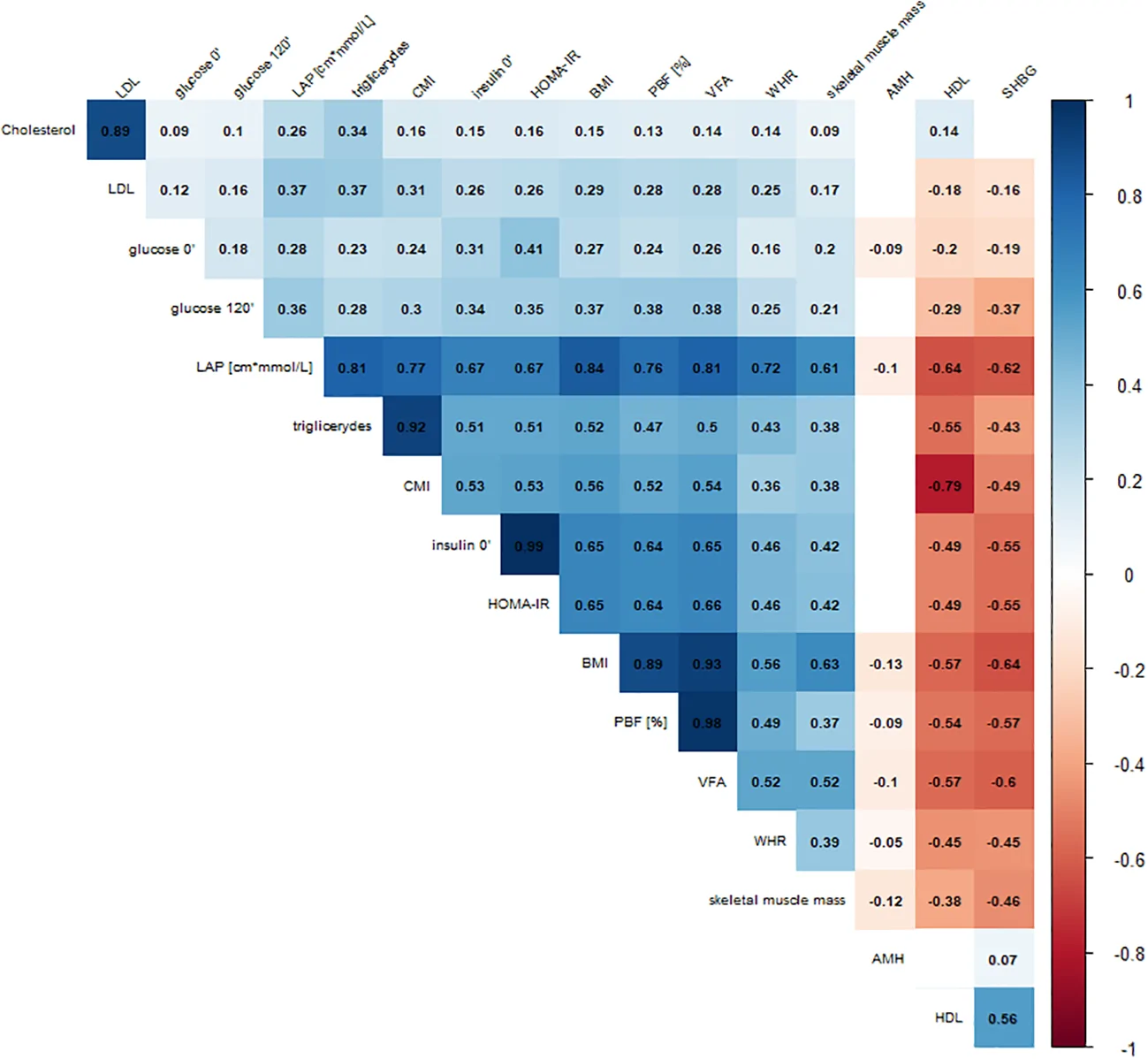
Correlation matrix of AMH and other variable data (only statistically significant correlations with *p* <.05 are shown). Heatmap showing correlations between AMH and selected variables. BMI, body mass index; WHR, waist–hip ratio; LDL, low-density lipoprotein; HDL, high-density lipoprotein; SHBG, sex-hormone binding globulin; AMH, anti-Müllerian, PBF–percent body fat body fat, VFA–visceral fat area, HOMA-IR, Homeostatic Model Assessment of Insulin Resistance, CMI–cardiometabolic index.

The metabolic parameters demonstrated several strong associations with the analyzed variables. Among them, LAP exhibited the strongest and most consistent correlations across the matrix. LAP was moderately or strongly positively correlated with CMI (*r* = .77), fasting insulin (*r* = .67), HOMA-IR (*r* = .67), percent body fat (*r* = .76), visceral fat area (*r* = .81), BMI (*r* = .84), WHR (*r* = .72), and skeletal muscle mass (*r* = .61). In contrast, LAP showed moderate negative associations with HDL (*r* = -.64) and SHBG concentrations (*r* = -.62).

### 3.2 The principal component analysis

Due to correlations among these metabolic parameters, PCA was conducted to reduce the number of variables for analysis. The following variables were included in the PCA analysis: BMI, WHR, triglycerides, cholesterol (LDL, HDL), SHBG, AMH, insulin (0’), glucose (0’, 120’), PBF, VFA, skeletal muscle mass, LAP, HOMA-IR, and CMI. The selection of dimensions for analysis was based on a scree plot, a standard technique in PCA (**Figure 2**).

**Figure 2:**
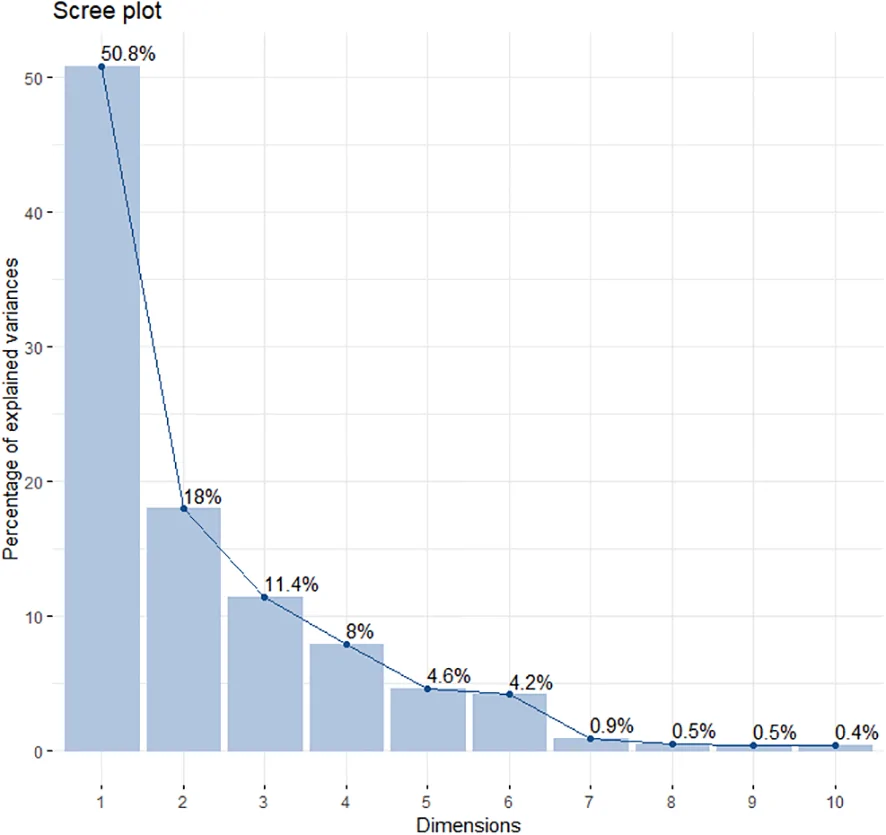
Scree plot.

Principal components were selected based on their significant contribution to explaining the variance of the analyzed parameters, with a 15% variance contribution threshold. On this basis, two principal components were identified, collectively explaining 68.8% of the total variance.

The first dimension consisted of triglyceride concentrations, and the second dimension – cholesterol levels. The factors describing the first (PC1) and second (PC2) dimension were further analyzed. The isolated factors explained 68.8% of the total variance of the data, with factor 1 explaining 50.8% and factor 2 - 18%. The remaining dimensions accounted for only 31.2% of the variance and were therefore not included in the description of the examined variables. The components relevant to dimensions 1 and 2 are shown in **Figure 3**.

**Figure 3:**
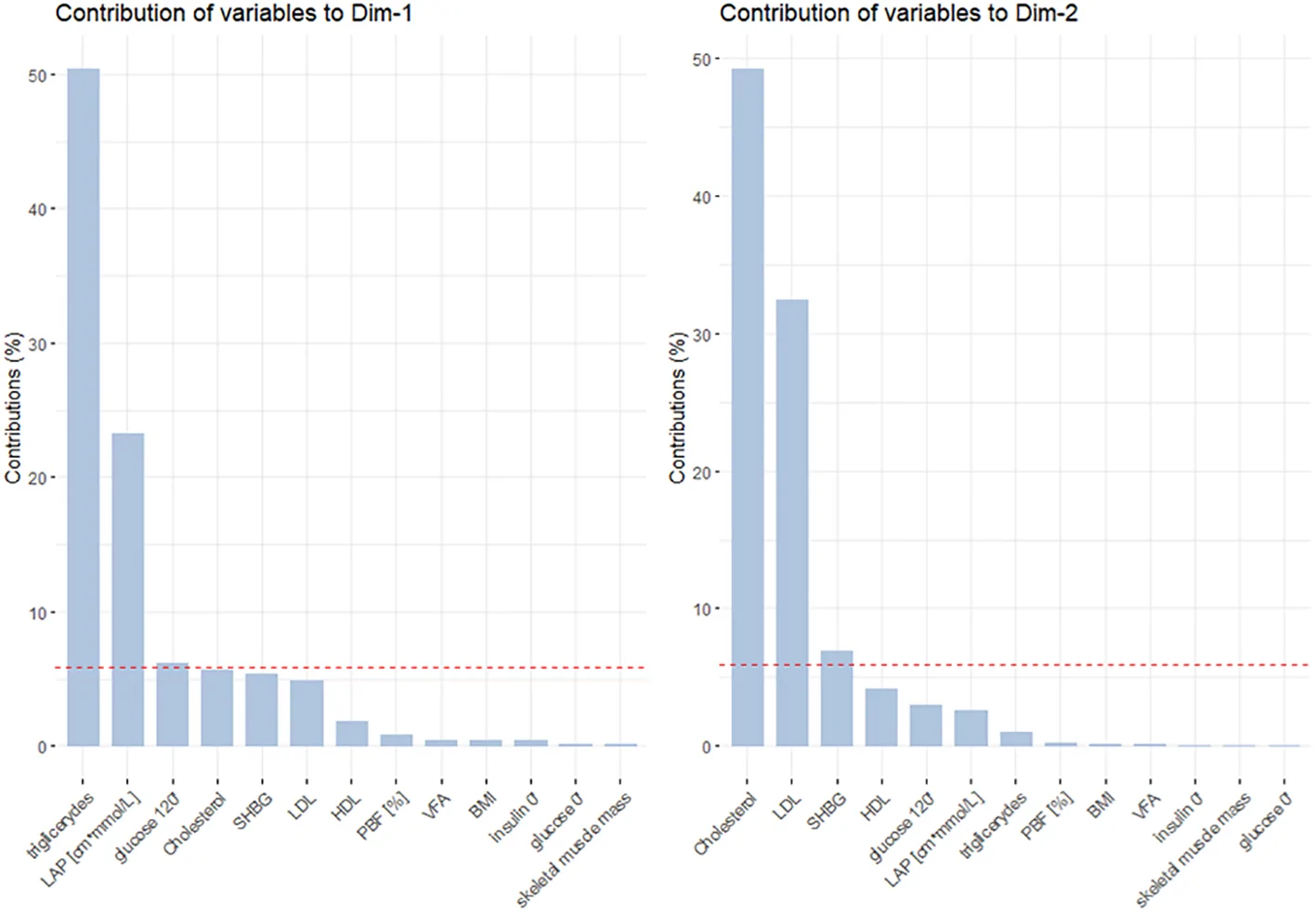
PCA of study variables. Scatter plot showing distribution of samples based on the first two principal components (PC1 and PC2). LAP, lipid accumulation product; SHBG, sex-hormone binding globulin; LDL, low-density lipoprotein; HDL, high-density lipoprotein; PBF, percent body fat body fat; VFA, visceral fat area, BMI, body mass index.

The correlation matrix shows irrelevant positive correlations between AMH and both principal components (PC1 and PC2) in the principal component analysis, as shown in **Figure 4**.

**Figure 4:**
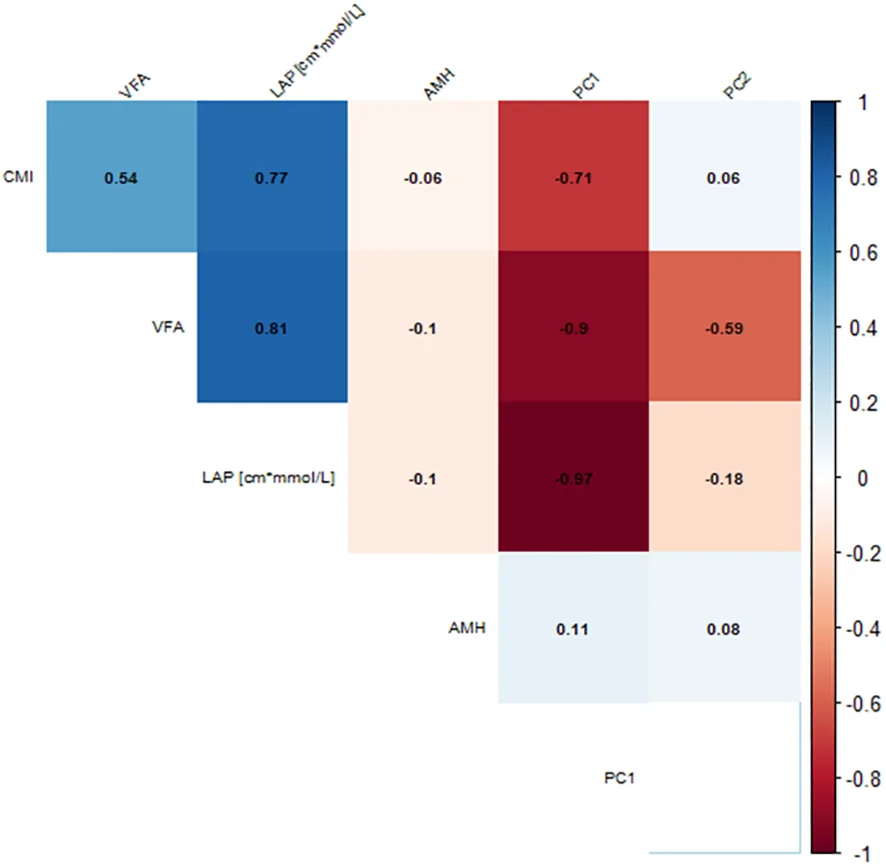
Correlation matrix of AMH and PCA (only statistically significant correlations with *p* <.05 are shown). Heatmap illustrating weak associations between AMH and the two principal components (PC1 and PC2).

### 3.3 Regression analysis

Univariable regression analyses highlighted significant association between AMH and PMOS phenotype. PMOS phenotypes 2, 3, and 4 were associated with significantly lower AMH concentrations compared to phenotype 1 (used as the reference category). Age, weight, BMI, hip and waist measurements, fasting glucose levels, VFA, skeletal muscle mass, and PC1 were significantly, but weakly associated with AMH concentrations. Detailed results of the univariable regression analyses are presented in **Table 2**.

**Table 2:** Univariable regression models assessing the association between AMH and examined variables.

Variable	Estimate (β)	Standard error	95% CI (lower)	95% CI (upper)	P-value
Age	-0.086	0.028	-0.14	-0.031	0.002
Weight	-0.025	0.007	-0.038	-0.012	0
Height	-0.002	0.022	-0.045	0.04	0.923
BMI	-0.073	0.019	-0.11	0.04	0
Waist	-0.022	0.009	-0.039	-0.005	0.01
Hip	-0.042	0.01	-0.062	-0.023	0
WHR	-1.346	1.596	-4.477	1.785	0.399
Triglycerides	-0.002	0.003	-0.007	0.004	0.505
Cholesterol	0.006	0.004	-0.003	0.014	0.173
LDL	0.005	0.005	-0.004	0.015	0.253
HDL	0.001	0.013	-0.025	0.027	0.939
SHBG	0.007	0.004	-0.001	0.016	0.095
Insulin 0’	-0.023	0.018	-0.058	0.013	0.215
Glucose 0’	-0.061	0.021	-0.102	-0.02	0.003
Glucose 120’	0	0.004	-0.007	0.008	0.903
PBF	-0.024	0.014	-0.052	0.003	0.081
VFA	-0.045	0.02	-0.084	-0.006	0.022
Skeletal muscle mass	-0.118	0.032	-0.18	-0.055	0
LAP	-0.008	0.004	-0.016	0	0.051
HOMA-IR	-0.134	0.081	-0.293	0.026	0.101
CMI	-0.236	0.209	-0.646	0.173	0.257
PMOS Phenotype 2	-3.61	0.34	-4.277	-2.943	0
PMOS Phenotype 3	-2.19	0.305	-2.789	-1.591	0
PMOS Phenotype 4	-0.8	0.395	-1.575	-0.025	0.043
PC1	0.008	0.003	0.002	0.015	0.013
PC2	0.018	0.01	-0.002	0.038	0.074

BMI, body mass index; WHR, waist–hip ratio; LDL, low-density lipoprotein; HDL, high-density lipoprotein; SHBG, sex-hormone binding globulin; AMH, anti-Müllerian, PBF–percent body fat body fat, VFA–visceral fat area, LAP, lipid accumulation product; HOMA-IR, Homeostatic Model Assessment of Insulin Resistance, CMI, cardiometabolic index. PMOS phenotype 1 was used as the reference category. Positive β values indicate a positive association with serum AMH concentration, whereas negative β values indicate a negative association. Statistical significance was defined as p <.05.

Multivariable linear regression analyses were performed to identify independent factors associated with serum AMH concentration (**Table 3**). BMI was negatively associated with serum AMH concentration in the models including height (β = −0.072, 95% CI −0.106 to −0.037, *p* < 0.001), fasting glucose (β = −0.071, 95% CI −0.108 to −0.034, *p* <.001), VFA (β = −0.166, 95% CI −0.262 to −0.070, *p* = .001), and PC1 (β = −0.101, 95% CI −0.166 to −0.037, *p* = .002). In contrast, BMI was not significantly associated with AMH in the models including body weight or skeletal muscle mass.

**Table 3:** Multivariable linear regression model of factors associated with serum AMH concentration.

Model	Predictor	β	SE	95% CI	P-value
1. Weight	Weight	−0.001	0.021	−0.043 to 0.040	0.949
	BMI	−0.068	0.060	−0.186 to 0.050	0.259
	Age	−0.047	0.025	−0.095 to 0.002	0.059
	PMOS phenotype 2	−3.294	0.344	−3.969 to −2.618	<0.001
	PMOS phenotype 3	−2.100	0.299	−2.686 to −1.514	<0.001
	PMOS phenotype 4	−0.810	0.393	−1.580 to −0.040	0.039
2. Height	Height	−0.003	0.020	−0.042 to 0.036	0.880
	BMI	−0.072	0.018	−0.106 to −0.037	<0.001
	Age	−0.045	0.025	−0.094 to 0.003	0.068
	PMOS phenotype 2	−3.295	0.344	−3.970 to −2.620	<0.001
	PMOS phenotype 3	−2.102	0.298	−2.687 to −1.517	<0.001
	PMOS phenotype 4	−0.811	0.393	−1.583 to −0.040	0.039
3. Fasting glucose	Fasting glucose	−0.023	0.020	−0.062 to 0.015	0.233
	BMI	−0.071	0.019	−0.108 to −0.034	<0.001
	Age	−0.032	0.025	−0.082 to 0.017	0.196
	PMOS phenotype 2	−3.302	0.346	−3.981 to −2.624	<0.001
	PMOS phenotype 3	−2.108	0.300	−2.698 to −1.519	<0.001
	PMOS phenotype 4	−0.702	0.399	−1.486 to 0.081	0.079
4. VFA	VFA	0.096	0.049	0.001 to 0.191	0.048
	BMI	−0.166	0.049	−0.262 to −0.070	0.001
	Age	−0.039	0.024	−0.087 to 0.009	0.113
	PMOS phenotype 2	−3.385	0.345	−4.061 to −2.708	<0.001
	PMOS phenotype 3	−2.082	0.301	−2.673 to −1.491	<0.001
	PMOS phenotype 4	−0.686	0.390	−1.452 to 0.080	0.079
5. Skeletal muscle mass	Skeletal muscle mass	−0.060	0.042	−0.143 to 0.022	0.152
	BMI	−0.042	0.026	−0.092 to 0.009	0.107
	Age	−0.048	0.025	−0.097 to 0.001	0.057
	PMOS phenotype 2	−3.298	0.345	−3.975 to −2.621	<0.001
	PMOS phenotype 3	−2.080	0.300	−2.669 to −1.490	<0.001
	PMOS phenotype 4	−0.898	0.391	−1.664 to −0.132	0.022
6. PC1	PC1	−0.006	0.006	−0.017 to 0.005	0.266
	BMI	−0.101	0.033	−0.166 to −0.037	0.002
	Age	−0.052	0.025	−0.101 to −0.002	0.040
	PMOS phenotype 2	−3.309	0.346	−3.987 to −2.630	<0.001
	PMOS phenotype 3	−2.084	0.298	−2.669 to −1.498	<0.001
	PMOS phenotype 4	−0.838	0.388	−1.599 to −0.077	0.031

AMH, anti-Müllerian hormone; BMI, body mass index; VFA, visceral fat area; PC1, first principal component; PCOS, polycystic ovary syndrome; β, unstandardized regression coefficient; SE, standard error; CI, confidence interval. Multivariable linear regression models included the indicated primary predictor and were adjusted for BMI, age, and PMOS phenotype. PMOS phenotype 1 was used as the reference category. Positive β values indicate a positive association with serum AMH concentration, whereas negative β values indicate a negative association. Statistical significance was defined as p <.05.

Age was not consistently associated with AMH across the models. A statistically significant inverse association was observed only in the PC1 model (β = −0.052, 95% CI −0.101 to −0.002, *p* = .040), whereas associations in the remaining models did not reach statistical significance.

PMOS phenotype was consistently associated with serum AMH concentration. Compared with phenotype 1, phenotype 2 and phenotype 3 were associated with significantly lower AMH concentrations in all models (all *p* <.001). Phenotype 4 was also associated with lower AMH concentrations in the models including body weight, height, skeletal muscle mass, and PC1, whereas this association did not reach statistical significance in the fasting glucose and VFA models.

Among the primary variables evaluated, VFA was the only parameter showing borderline statistically significant independent association with serum AMH concentration. Higher VFA was associated with higher AMH concentrations (β = 0.096, 95% CI 0.001–0.191, *p* = .048), despite the inverse association between BMI and AMH in the same model. Neither body weight, height, fasting glucose, skeletal muscle mass, nor PC1 was independently associated with serum AMH concentration.

## 4 Discussion

In this study, we found that the serum concentration of AMH marginally correlated with parameters of adiposity, including PBF, VFA, CMI, and LAP. Despite statistical significance, given the low correlation coefficient values, we consider them negligible in a clinical setting.

The results obtained in this study suggest that while visceral obesity does influence fertility among women, both with PMOS and without this syndrome, it might not be related to the AMH concentration specifically. This can be explained by mechanisms regulating oocyte number and AMH release being partially independent of systemic adiposity, or by the metabolic effect not specifically influencing the oocyte population (33, 34). While it is known that visceral fat influences fertility by disrupting ovulation, as well as the quality of oocytes or the metabolic environment of endometrial tissue (35, 36), it might not correlate with AMH concentration as anticipated.

There is limited information on the effect of visceral obesity on the AMH serum concentration. However, our findings are consistent with other research projects suggesting either no correlation between metabolic markers and AMH or a negative association between AMH and metabolic markers. Most studies indicate that AMH is more influenced by BMI and insulin resistance than by the cardiometabolic variables themselves (31, 37).

However, in this study, indicators of adiposity, such as LAP, CMI, PBF, VFA and WHR, showed statistically significant correlations with metabolic and anthropometric parameters. The strongest associations were observed for LAP, which was positively correlated with CMI, fasting insulin, HOMA-IR, percent body fat, visceral fat area, BMI, WHR, and skeletal muscle mass. Similarly, CMI was positively correlated with HOMA-IR (*r* = .53), and BMI (*r* = .54), whereas indicators directly reflecting body fat, such as VFA and PBF, were strongly associated with BMI (*r* = .93 and *r* = .89, respectively) and insulin resistance parameters (*r* = .66 and *r* = .64, respectively). Therefore, our data confirm they are effective markers of metabolic syndrome and obesity-related diseases, with high diagnostic accuracy (AUC ~0.82) (31).

On the other hand, anthropometric indicators such as BMI, PBF, and WHR also showed significant correlations with each other and with metabolic parameters, but these correlations were weaker than those of composite indices (LAP, CMI). This is because BMI does not reflect body fat distribution, whereas WHR considers only body proportions and lacks a metabolic component. In contrast, LAP and CMI integrate both anthropometric and biochemical data, which increases their ability to identify metabolic disorders. In turn, this study showed a negative correlation between the analyzed indicators and HDL. Population studies have shown that increases in indicators such as LAP are associated with decreases in HDL and increases in cardiovascular risk (38).

The strong correlations between adiposity and metabolic indices (LAP, CMI, VFA, PBF, WHR) observed in this study could be explained by the pathophysiology of polyendocrine metabolic ovarian syndrome, in which abnormal fat distribution and insulin resistance play a key role in its development. Visceral adipose tissue is characterized by high metabolic activity and leads to increased release of free fatty acids and proinflammatory adipokines, which directly contribute to the development of insulin resistance and hyperinsulinemia, and this is the cause of excessive production of androgens by the ovaries (39, 40). The strong correlations of LAP and CMI with the rest of adiposity and metabolic indices shows they might be better markers of metabolic disorders in PMOS women than BMI (41). In turn, the strong associations between VFA and WHR and metabolic parameters might result from hyperandrogenism, which, in normal-weight PMOS women, is associated with preferential intra-abdominal fat deposition. It could explain why indicators such as WHR and VFA are more useful in this patient group (42). Others also place greater emphasis on age as the primary factor affecting hormone concentration (43). The heterogeneity observed across studies may stem from differences in cohort characteristics, including age distribution, ethnic background, the proportion of participants with and without PMOS, as well as variations in study design and methodology.

The application of PCA has brought a clinical perspective, as it allows for the consideration of interrelated metabolic parameters as shared dimensions rather than analyzing each variable independently. The first component primarily reflected the shared variance of triglycerides and LAP, while the second was mainly associated with total cholesterol and LDL. This approach enables a broader assessment of the relationships between complex metabolic profiles and AMH concentrations, accounting for the co-occurrence and interdependencies of individual parameters. It suggests that lipid profile is more crucial in the characterization of the group than the body mass alone. A substantial contribution of LAP to PC1 confirms its utility as a marker of metabolic dysfunction, closely related to triglyceride metabolism in the analyzed cohort (32, 44, 45). Aside from total cholesterol and LDL fraction, another useful determinant in Dim-2 was SHBG. In PMOS, the role of SHBG extends beyond its function in transporting androgens, making it a sensitive marker of metabolic disturbances (46). The co-occurrence of these variables along one dimension suggests a shared pathophysiological basis, likely linked to their metabolic function in the liver. In PMOS, compensatory hyperinsulinemia not only disrupts lipid pathways but also directly inhibits hepatic SHBG synthesis as reflected in the structure of Dim-2 (46). Direct separation of the triglyceride axis in Dim-1 from the cholesterol-SHBG axis (Dim-2) might suggest different metabolic phenotypes in the examined group, from patients with dominating hypertriglyceridemia and insulin resistance, to patients with LDL profile disturbances and low levels of SHBG. This analysis might confirm that cardiovascular risk assessment in women with PMOS requires examination of SHBG and other derived indicators, as these determinants provide crucial information about the organism’s homeostasis.

### 4.1 Strengths and limitations

The present study has several strengths. Its large sample size allows for more reliable generalization of the findings, thereby making the results more representative and less susceptible to random variation. AMH was assessed within a strictly defined and narrow reproductive age range and in the early follicular phase of the menstrual cycle. Its scarce influence as a confounding factor, together with BMI, has been proved in regression analysis. Moreover, including adiposity and metabolic markers rarely assessed in the PMOS population - such as LAP, VFA, and CMI - substantially strengthens the study’s scientific contribution.

One limitation of the study is its retrospective design, which precludes determining the direction of associations and prevents any inference about causal relationships. Additionally, the lack of detailed information on participants’ lifestyle factors and other health-related behaviors limits the ability to fully control potential confounders associated with metabolic parameters. Another limitation is the use of methods based on bioelectrical impedance, which- although practical and widely applied- provides less precise estimates of body fat content, particularly visceral adipose tissue, compared with more accurate imaging methods, e.g. DXA or CT/MRI.

### 4.2 Future research

Based on regression analysis showing PMOS phenotype as a predictor of AMH, an interesting direction of future research would be to stratify women with PMOS into phenotypic subgroups according to the Rotterdam criteria (phenotypes A-D) to determine whether the above metabolic parameters vary systematically across these clinical entities. In addition, CT or MRI could be used to measure visceral fat to obtain more accurate data.

## 5 Conclusions

To conclude, in this large cohort of women with PMOS, circulating AMH concentrations showed negligible associations with measures of adiposity and visceral adiposity. In contrast, adiposity indices, particularly LAP and CMI, demonstrated strong associations with metabolic parameters, reflecting their composite construction and their relationship with cardiometabolic dysfunction. These findings suggest that circulating AMH may not be a useful surrogate marker of visceral adiposity in women with PMOS; however, the cross-sectional design precludes conclusions regarding causality.

## Data Availability

The datasets used and analyzed during the current study are available from the corresponding author upon request. Requests to access these datasets should be directed to karolina.kowalczyk@sum.edu.pl.

## References

[1] Teede HJ Khomami MB Morman R Laven JSE Joham AE Costello MF . Polyendocrine metabolic ovarian syndrome, the new name for polycystic ovary syndrome: a multistep global consensus process. Lancet. (2026) 407:2329–39. doi: 10.1016/S0140-6736(26)00717-8 42119588

[2] Singh S Pal N Shubham S Sarma DK Verma V Marotta F . Polycystic ovary syndrome: etiology, current management, and future therapeutics. J Clin Med. (2023) 12:1454. doi: 10.3390/jcm12041454 36835989 PMC9964744

[3] Teede H Deeks A Moran L . Polycystic ovary syndrome: a complex condition with psychological, reproductive and metabolic manifestations that impacts on health across the lifespan. BMC Med. (2010) 8:41. doi: 10.1186/1741-7015-8-41 20591140 PMC2909929

[4] Teede HJ Tay CT Laven JJE Dokras A Moran LJ Piltonen TT . Recommendations from the 2023 international evidence-based guideline for the assessment and management of polycystic ovary syndrome. J Clin Endocrinol Metab. (2023) 108:2447–69. doi: 10.1210/clinem/dgad463 37580314 PMC10505534

[5] Łebkowska A Kowalska I . Anti-Müllerian hormone and polycystic ovary syndrome. Endokrynol Pol. (2017) 68(1):74–8. doi: 10.5603/EP.a2016.0065 27918066

[6] Rudnicka E Kunicki M Calik-Ksepka A Suchta K Duszewska A Smolarczyk K . Anti-Müllerian hormone in pathogenesis, diagnostic and treatment of PCOS. Int J Mol Sci. (2021) 22:12507. doi: 10.3390/ijms222212507 34830389 PMC8619458

[7] Shi W Zhao Q Zhao X Xing C He B . Analysis of endocrine and metabolic indexes in non-obese patients with polycystic ovary syndrome and its compare with obese patients. Diabetes Metab Syndr Obes Targets Ther. (2021) 14:4275–81. doi: 10.2147/DMSO.S329108 34703260 PMC8526511

[8] Fonseca HP Brondi RS Piovesan FX Miklos TG Aldrighi JM . Anti-Mullerian hormone and insulin resistance in polycystic ovary syndrome. Gynecol Endocrinol. (2014) 30:667–70. doi: 10.3109/09513590.2014.920004 24898132

[9] Cui Y Shi Y Cui L Han T Gao X Chen ZJ . Age-specific serum antimüllerian hormone levels in women with and without polycystic ovary syndrome. Fertil Steril. (2014) 102:230–236.e2. doi: 10.1016/j.fertnstert.2014.03.032 24746743

[10] Zhao H Zhou D Liu C Zhang L . The relationship between insulin resistance and obesity and serum anti-mullerian hormone level in Chinese women with polycystic ovary syndrome: a retrospective, single-center cohort study. Int J Womens Health. (2023) 15:151–66. doi: 10.2147/IJWH.S393594 36778752 PMC9911904

[11] Zhang M Liu X Xu X Li J Bu Z Yang Q . The reference value of anti-müllerian hormone to diagnose polycystic ovary syndrome is inversely associated with BMI: a retrospective study. Reprod Biol Endocrinol. (2023) 21:15. doi: 10.1186/s12958-023-01064-y 36726106 PMC9890853

[12] Skałba P Cygal A Madej P Dąbkowska-Huć A Sikora J Martirosian G . Is the plasma anti-müllerian hormone (AMH) level associated with body weight and metabolic, and hormonal disturbances in women with and without polycystic ovary syndrome? Eur J Obstet Gynecol Reprod Biol. (2011) 158:254–9. doi: 10.1016/j.ejogrb.2011.06.006 21752527

[13] Hwang YI Sung NY Koo HS Cha SH Park CW Kim JY . Can high serum anti-müllerian hormone levels predict the phenotypes of polycystic ovary syndrome (PCOS) and metabolic disturbances in PCOS patients? Clin Exp Reprod Med. (2013) 40:135. doi: 10.5653/cerm.2013.40.3.135 24179872 PMC3811721

[14] Feldman RA O’Neill K Butts SF Dokras A . Antimüllerian hormone levels and cardiometabolic risk in young women with polycystic ovary syndrome. Fertil Steril. (2017) 107:276–81. doi: 10.1016/j.fertnstert.2016.10.009 27842995

[15] Lucas E Aronne LJ . Is it time to define obesity by body composition and not solely body mass index? J Clin Endocrinol Metab. (2025) 110:e1278–9. doi: 10.1210/clinem/dgae473 38994573

[16] Oliveros E Somers VK Sochor O Goel K Lopez-Jimenez F . The concept of normal weight obesity. Prog Cardiovasc Dis. (2014) 56:426–33. doi: 10.1016/j.pcad.2013.10.003 24438734

[17] Shah RV Murthy VL Abbasi SA Blankstein R Kwong RY Goldfine AB . Visceral adiposity and the risk of metabolic syndrome across body mass index. JACC Cardiovasc Imaging. (2014) 7:1221–35. doi: 10.1016/j.jcmg.2014.07.017 25440591 PMC4268163

[18] Carvalho JB De Andrade GKP Do Nascimento LA Golin N Rodrigues ALCC Suiter E . Visceral fat area measured by electrical bioimpedance as an aggravating factor of COVID-19: a study on body composition. BMC Infect Dis. (2023) 23:826. doi: 10.1186/s12879-023-08833-5 38001401 PMC10675966

[19] Khalil S Mohktar M Ibrahim F . The theory and fundamentals of bioimpedance analysis in clinical status monitoring and diagnosis of diseases. Sensors. (2014) 14:10895–928. doi: 10.3390/s140610895 24949644 PMC4118362

[20] Silva AM Campa F Stagi S Gobbo LA Buffa R Toselli S . The bioelectrical impedance analysis (BIA) international database: aims, scope, and call for data. Eur J Clin Nutr. (2023) 77:1143–50. doi: 10.1038/s41430-023-01310-x 37532867

[21] Zakeri A Ebadinejad A Rahmati M Momenan AA Niroomand M Valizadeh M . Body composition analysis in women with polycystic ovary syndrome: a cross-sectional study from the Tehran Lipid and Glucose Study (TLGS). BMC Endocr Disord. (2024) 24:251. doi: 10.1186/s12902-024-01783-2 39558339 PMC11575009

[22] Zhang H Wang W Zhao J Jiao P Zeng L Zhang H . Relationship between body composition, insulin resistance, and hormonal profiles in women with polycystic ovary syndrome. Front Endocrinol. (2023) 13:1085656. doi: 10.3389/fendo.2022.1085656 36699018 PMC9869160

[23] Chitme HR Al Azawi EAK Al Abri AM Al Busaidi BM Salam ZKA Al Taie MM . Anthropometric and body composition analysis of infertile women with polycystic ovary syndrome. J Taibah Univ Med Sci. (2017) 12:139–45. doi: 10.1016/j.jtumed.2016.11.005 31435228 PMC6694878

[24] Achamrah N Colange G Delay J Rimbert A Folope V Petit A . Comparison of body composition assessment by DXA and BIA according to the body mass index: a retrospective study on 3655 measures. PloS One. (2018) 13:e0200465. doi: 10.1371/journal.pone.0200465 30001381 PMC6042744

[25] Ebrahimi M Seyedi SA Nabipoorashrafi SA Rabizadeh S Sarzaeim M Yadegar A . Lipid accumulation product (LAP) index for the diagnosis of nonalcoholic fatty liver disease (NAFLD): a systematic review and meta-analysis. Lipids Health Dis. (2023) 22:41. doi: 10.1186/s12944-023-01802-6 36922815 PMC10015691

[26] Kahn HS . The “lipid accumulation product” performs better than the body mass index for recognizing cardiovascular risk: a population-based comparison. BMC Cardiovasc Disord. (2005) 5:26. doi: 10.1186/1471-2261-5-26 16150143 PMC1236917

[27] Shi WR Wang HY Chen S Guo XF Li Z Sun YX . Estimate of prevalent diabetes from cardiometabolic index in general Chinese population: a community-based study. Lipids Health Dis. (2018) 17:236. doi: 10.1186/s12944-018-0886-2 30314516 PMC6186046

[28] The Rotterdam ESHRE/ASRM-sponsored PCOS consensus workshop group . Revised 2003 consensus on diagnostic criteria and long-term health risks related to polycystic ovary syndrome (PCOS). Hum Reprod. (2004) 19:41–7. doi: 10.1093/humrep/deh098 14688154

[29] Hatch R Rosenfield RL Kim MH Tredway D . Hirsutism: implications, etiology, and management. Am J Obstet Gynecol. (1981) 140:815–30. doi: 10.1016/0002-9378(81)90746-8 7258262

[30] Lizneva D Suturina L Walker W Brakta S Gavrilova-Jordan L Azziz R . Criteria, prevalence, and phenotypes of polycystic ovary syndrome. Fertil Steril. (2016) 106:6–15. doi: 10.1016/j.fertnstert.2016.05.003 27233760

[31] Tamini S Bondesan A Caroli D Sartorio A . The lipid accumulation product index (LAP) and the cardiometabolic index (CMI) are useful for predicting the presence and severity of metabolic syndrome in adult patients with obesity. J Clin Med. (2024) 13:2843. doi: 10.3390/jcm13102843 38792386 PMC11122168

[32] Seyedi SA Nabipoorashrafi SA Amin Afshari S Mansouri A Alizadegan D Hobaby S . Lipid accumulation product index as a marker of metabolic syndrome in women with polycystic ovary syndrome: a systematic review and meta‐analysis. Endocrinol Diabetes Metab. (2025) 8:e70078. doi: 10.1002/edm2.70078 40908595 PMC12411561

[33] Kloos J Coyne K Weinerman R . The relationship between anti‐müllerian hormone, body mass index and weight loss: a review of the literature. Clin Obes. (2022) 12:e12559. doi: 10.1111/cob.12559 36181300 PMC9787654

[34] Zeng X Huang Y Zhang M Chen Y Ye J Han Y . Anti-müllerian hormone was independently associated with central obesity but not with general obesity in women with PCOS. Endocr Connect. (2022) 11:e210243. doi: 10.1530/EC-21-0243 34822342 PMC8789017

[35] Anaya ES De Groot EL Lydon JP Pangas SA Hartig SM . Contributions of white adipose tissue to energy requirements for female reproduction. Trends Endocrinol Metab. (2024) 35:809–20. doi: 10.1016/j.tem.2024.04.012 38749883 PMC11387141

[36] Skowrońska M Pawłowski M Buczyńska A Wiatr A Dyszkiewicz A Wenta A . The relationship between body composition parameters and the intake of selected nutrients, and serum anti-müllerian hormone (AMH) levels in the context of ovulatory infertility. Nutrients. (2024) 16:4149. doi: 10.3390/nu16234149 39683543 PMC11644442

[37] Li X Tang Q Feng Y Zhang Y Tian W Zhang H . The relationship between anti-müllerian hormone and body composition components in patients with polycystic ovary syndrome of reproductive age. Reprod Sci. (2024) 31:2843–8. doi: 10.1007/s43032-024-01561-4 38653855

[38] Cartolano FDC Pappiani C Freitas MCP Figueiredo Neto AM Carioca AAF Damasceno NRT . Is lipid accumulation product associated with an atherogenic lipoprotein profile in Brazilian subjects? Arq Bras Cardiol. (2018) 110(4):339–47. doi: 10.5935/abc.20180054 29791572 PMC5941956

[39] Bulu A Onalan E Yakar B Bulu G Onalan Yıldırım S Uğur K . Investigation of inflammatory markers in patients with polycystic ovary syndrome receiving and not receiving metformin treatment. Med (Kaunas). (2025) 61:1012. doi: 10.3390/medicina61061012 40572700 PMC12195471

[40] Zhao H Zhang J Cheng X Nie X He B . Insulin resistance in polycystic ovary syndrome across various tissues: an updated review of pathogenesis, evaluation, and treatment. J Ovarian Res. (2023) 16:9. doi: 10.1186/s13048-022-01091-0 36631836 PMC9832677

[41] Liu Q Xie Y Qu L Zhang M Mo Z . Dyslipidemia involvement in the development of polycystic ovary syndrome. Taiwan J Obstet Gynecol. (2019) 58:447–53. doi: 10.1016/j.tjog.2019.05.003 31307731

[42] Dumesic DA Akopians AL Madrigal VK Ramirez E Margolis DJ Sarma MK . Hyperandrogenism accompanies increased intra-abdominal fat storage in normal weight polycystic ovary syndrome women. J Clin Endocrinol Metab. (2016) 101:4178–88. doi: 10.1210/jc.2016-2586 27571186 PMC5095243

[43] Bahadur A Verma N Mundhra R Chawla L Ajmani M Sri MS . Correlation of homeostatic model assessment-insulin resistance, anti-mullerian hormone, and BMI in the characterization of polycystic ovary syndrome. Cureus. (2021) 13(6):e16047. doi: 10.7759/cureus.16047 34336524 PMC8321419

[44] Wiltgen D Benedetto IG Mastella LS Spritzer PM . Lipid accumulation product index: a reliable marker of cardiovascular risk in polycystic ovary syndrome. Hum Reprod. (2009) 24:1726–31. doi: 10.1093/humrep/dep072 19329517

[45] Liu T Bo W Cao Q Wang R Qiao X Zhong Y . The accuracy of lipid accumulation product to predict metabolic syndrome in PCOS: a meta-analysis and comparative analysis with other indicators. Front Endocrinol. (2026) 17:1709946. doi: 10.3389/fendo.2026.1709946 42064769 PMC13124632

[46] Xing C Zhang J Zhao H He B . Effect of sex hormone-binding globulin on polycystic ovary syndrome: mechanisms, manifestations, genetics, and treatment. Int J Womens Health. (2022) 14:91–105. doi: 10.2147/IJWH.S344542 35140526 PMC8818772

